# Cardiac hypertrophy limits infarct expansion after myocardial infarction in mice

**DOI:** 10.1038/s41598-018-24525-6

**Published:** 2018-04-17

**Authors:** Siiri E. Iismaa, Ming Li, Scott Kesteven, Jianxin Wu, Andrea Y. Chan, Sara R. Holman, John W. Calvert, Ahtesham ul Haq, Amy M. Nicks, Nawazish Naqvi, Ahsan Husain, Michael P. Feneley, Robert M. Graham

**Affiliations:** 1 Division of Molecular Cardiology and Biophysics, Victor Chang Cardiac Research Institute, Sydney, NSW 2010 Australia; 2 St Vincent’s Clinical School, University of New South Wales, Sydney, NSW 2052 Australia; 3 Cardiac Regeneration Research Institute, Wenzhou Medical University, Wenzhou, 325035 China; 4 Department of Medicine, Emory University School of Medicine, Atlanta, GA 30322 USA; 5 Department of Surgery, Emory University School of Medicine, Atlanta, GA 30308 USA

## Abstract

We have previously demonstrated that adult transgenic C57BL/6J mice with CM-restricted overexpression of the dominant negative *W*^*v*^ mutant protein (dn-c-kit-Tg) respond to pressure overload with robust cardiomyocyte (CM) cell cycle entry. Here, we tested if outcomes after myocardial infarction (MI) due to coronary artery ligation are improved in this transgenic model. Compared to non-transgenic littermates (NTLs), adult male dn-c-kit-Tg mice displayed CM hypertrophy and concentric left ventricular (LV) hypertrophy in the absence of an increase in workload. Stroke volume and cardiac output were preserved and LV wall stress was markedly lower than that in NTLs, leading to a more energy-efficient heart. In response to MI, infarct size in adult (16-week old) dn-c-kit-Tg hearts was similar to that of NTL after 24 h but was half that in NTL hearts 12 weeks post-MI. Cumulative CM cell cycle entry was only modestly increased in dn-c-kit-Tg hearts. However, dn-c-kit-Tg mice were more resistant to infarct expansion, adverse LV remodelling and contractile dysfunction, and suffered no early death from LV rupture, relative to NTL mice. Thus, pre-existing cardiac hypertrophy lowers wall stress in dn-c-kit-Tg hearts, limits infarct expansion and prevents death from myocardial rupture.

## Introduction

Global functional inactivation (by ~95%) of c-kit (*W/Wv* mice), the receptor for stem cell factor, prevents the normal developmental acquisition of CM terminal differentiation in the adult animal^1^. At baseline, the cardiac phenotype in *W/Wv* mice is virtually indistinguishable from that of their congenic wild type littermates. However, when subjected to increased pressure overload, cardiac enlargement in *W/Wv* mice occurs mainly through CM hyperplasia rather than by CM hypertrophy^1^. Similarly, CM-restricted overexpression of the dominant-negative *Wv* c-kit mutant protein, c-kitT660M, in mice (dn-c-kit-Tg mice) has no effect on CM proliferation in untreated adult (13-week-old) mice but results in CM cell cycle entry in response to pressure overload, with BrdU^+^, H3P^+^ and Aurora B^+^ CMs readily apparent in left ventricles one week after pressure overload^1^. Thus, expression of dn-c-kit in CMs after birth is sufficient for pressure overload-induced cell cycle entry of adult CMs^1^.

*W/Wv* mice are poorly suited for studies of cardiac regeneration post-MI because concomitant inactivation of c-kit on endothelial progenitor cells diminishes the ability of the heart to mount a robust angiogenic response following injurious loss of myocardium^2^. The CM-restricted dn-c-kit-Tg model should, however, lend itself to the study of cardiac regeneration in adult hearts because the heart is composed of CMs that are quiescent at baseline but can be stimulated to proliferate^1^, and the endothelial progenitor cells are unaffected. Here we describe the baseline cardiac phenotype of the dn-c-kit-Tg mouse and show that compared to NTL controls, CM-specific overexpression of dn-c-kit resulted in CM hypertrophy in adulthood. Afterload (left ventricular systolic pressure) was unaltered in this model, indicating the cardiac hypertrophy was primary, and was associated with preserved stroke volume and cardiac output. Moreover, wall stress at baseline was lower in dn-c-kit-Tg mice as a result of increased LV wall thickness and a commensurate decrease in LV chamber diameter, consistent with a more energy-efficient heart. Previous clinical studies have suggested that cardiac hypertrophy is protective of infarct expansion, but it was unclear if the hypertrophy associated with improved outcomes was pre-existing or was the result of post-infarct remodelling^3^. We explored the impact of the phenotypic differences in the cellular and structural properties of the dn-c-kit-Tg heart on the response to permanent coronary artery ligation. Contrary to the response observed with pressure overload^1^, there was only a modest increase in CM cell cycle entry in dn-c-kit-Tg hearts after MI. However, relative to NTL mice, dn-c-kit-Tg mice were much more resistant to MI-induced infarct expansion, adverse cardiac remodelling and dysfunction, and, unlike NTL mice, suffered no early death from LV rupture. Thus, although MI did not trigger substantial CM cell cycle entry in dn-c-kit-Tg mice, the pre-existing hypertrophy lowers wall stress, limits infarct expansion and prevents death from myocardial rupture.

## Results

### CM-specific overexpression of dn-c-kit results in primary CM hypertrophy leading to a thicker LV wall, a smaller LV cavity and higher ejection fraction

**Figure 1:**
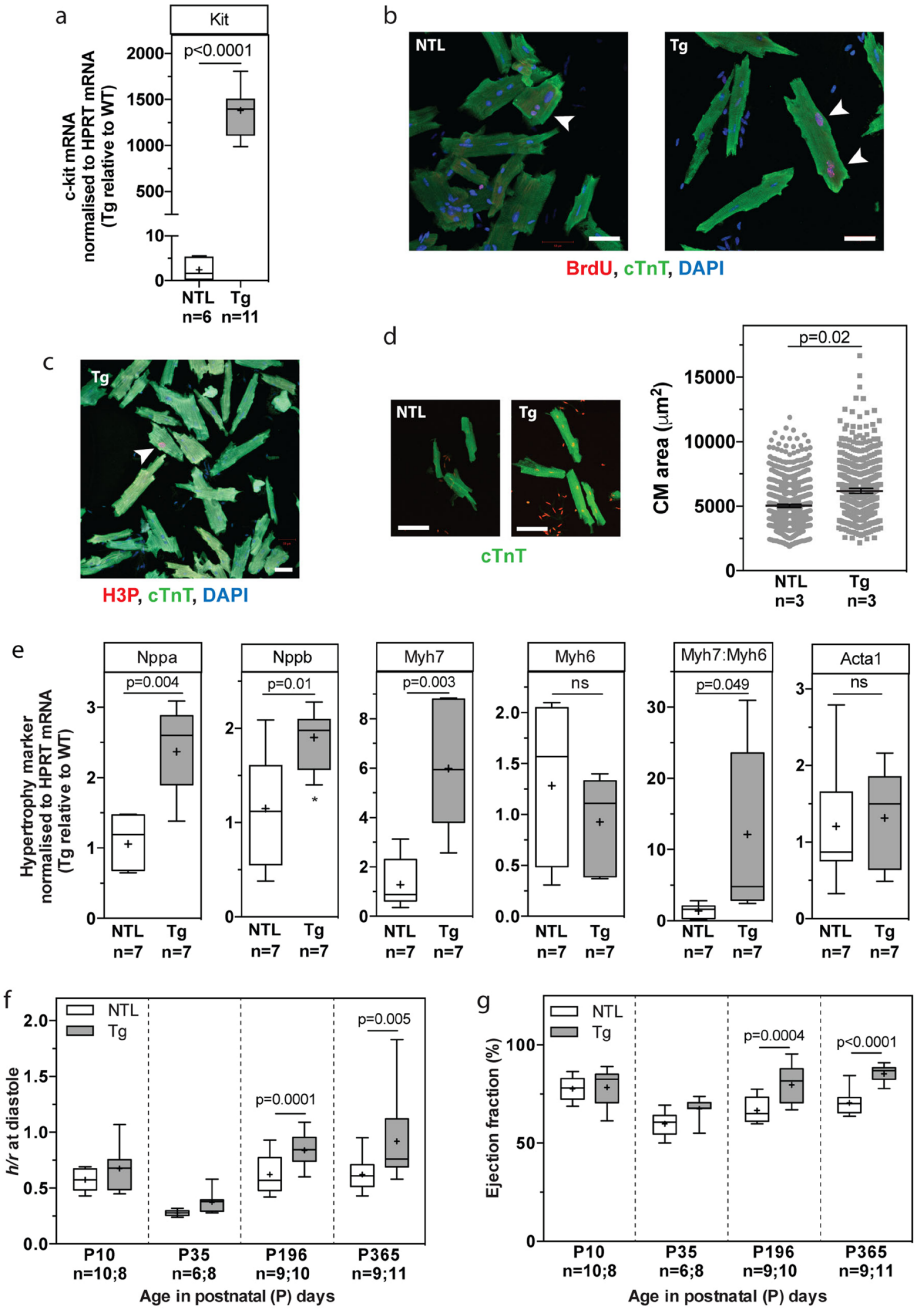
CM-specific overexpression of dn-c-kit resulted in CM hypertrophy, leading to a thicker LV wall, a smaller LV cavity and higher ejection fraction. (**a**) Expression of *Kit* (c-Kit) mRNA in male P196 dn-c-kit-Tg (Tg; n = 11) and NTL (n = 6) hearts; differences analysed by Student’s *t*-test. **(b**,**c**) Examples (white arrowheads) of BrdU^+^/cTnT^+^ (**b**) or H3P^+^/cTnT^+^ (**c**) CMs isolated from P121 Tg (**b**, right panel, and **c**) or NTL (**b**, left panel) hearts after 9 days of BrdU (10 mg/kg/day) infusion by osmotic mini-pump. Scale bar: 50 μm. (**d**) Left panels, representative photographs, and right panel, quantitation of surface area showing hypertrophy of isolated CMs from P121 Tg (682 CMs analysed from n = 3 mice) relative to NTL hearts (1015 CMs analysed from n = 3 mice); differences analysed by Student’s *t*-test. Scale bar: 100 μm. (**e**) Abundance of *Myh6* (encoding α-MHC) and *Acta1* (α-skeletal actin) mRNAs was similar, while abundance of other mRNAs (*Nppa* (ANP), *Nppb* (BNP), *Myh*7 (β-MHC)) and the *Myh*7*:Myh6* (β-:α-MHC) ratio were significantly increased in male P196 dn-c-kit-Tg relative to NTL hearts (n = 7); differences analysed by Student’s *t*-test. (**f**) Increased LV wall thickness to chamber radius (*h/r*) ratio at end-diastole in P ≥ 196 dn-c-kit-Tg (n = 10–11) relative to NTL (n = 9) hearts; differences analysed by two-way ANOVA with Tukey’s multiple comparison test. (**g**) Increased ejection fraction in P ≥ 196 dn-c-kit-Tg (n = 10–11) relative to NTL (n = 9) hearts; differences analysed by two-way ANOVA with Tukey’s multiple comparison test.

CM-specific overexpression of dn-c-kit (Fig. 1a) did not alter the rate or extent of body growth from birth to one-year-of-age (Table 1). At postnatal day 10 (P10), soon after the end of the neonatal period, cardiac growth, structure and function were not different between NTL and dn-c-kit-Tg mice (Tables 1 and 2). By P35, dn-c-kit-Tg hearts were ~1.2-fold heavier than NTL hearts (p = 0.003), and this was almost entirely due to an increase in left ventricular (LV) weight (p = 0.008) and wall thickness (p = 0.02) (Tables 1 and 2). This difference in heart weight was maintained through to one-year-of-age (Table 1). To assess CM cell cycle entry in adulthood, BrdU was delivered to adult (P112) mice for nine days by implanted osmotic mini-pumps, followed by isolation of CMs. A higher percentage of dn-c-kit-Tg CMs were observed in cell cycle (BrdU^+^/cTnT^+^ CMs, p = 0.009) (Fig. 1b, Table 3) and in metaphase (H3P^+^/cTnT^+^ CMs) (Fig. 1c, Table 3), but not in anaphase or cytokinesis (AurB^+^/cTnT^+^), relative to NTL CMs (Table 3). This increase in cell cycle activity in dn-c-kit-Tg CMs was insufficient to significantly impact nuclear ploidy (Supplementary Fig. S1, Table 3), nucleation state, or the number of adult CMs in the heart, relative to NTL (Table 3). There was also no evidence in P112 NTL or dn-c-kit-Tg hearts of myofibrillar disarray or CM apoptosis by TUNEL staining (Supplementary Fig. S2, Table 3). CM-specific expression of dn-c-kit did, however, result in a greater CM area relative to NTL (p < 0.0001) in P112 hearts (Fig. 1d, Table 3). Evaluation of hypertrophy marker genes indicated activation of the fetal gene program with enhanced abundance of *Nppa* (encoding ANP, p = 0.004), *Nppb* (encoding BNP, p = 0.01), and *Myh*7 (encoding β-MHC, p = 0.003) mRNAs in dn-c-kit-Tg relative to NTL adult hearts. However, there was no difference in abundance of *Myh6* (encoding α-MHC, p = ns) mRNA, which typically decreases with pathological cardiac hypertrophy^4^, so that the *Myh*7*:Myh6* ratio was markedly higher (by 12-fold; p = 0.049) in dn-c-kit-Tg hearts relative to NTL hearts (Fig. 1e). There was no difference in *Acta1* (encoding α-skeletal actin, p = ns) mRNA levels (Fig. 1e).

**Table 1:** NTL and dn-c-kit-Tg cardiac morphometry during postnatal and adult development.

Parameter	Genotype	P10 *(n)*	P18 *(n)*	P35 *(n)*	P112 (*n*)	P196 *(n)*	P365 *(n)*
BW (g)	NTL	4.68 ± 0.11 *(9)*	8.69 ± 0.28 *(5)*	18.4 ± 0.3 *(10)*	30.96 ± 0.8 (*7*)	31.64 ± 0.86 *(9)*	33.2 ± 0.72 *(9)*
	Tg	4.87 ± 0.13 *(6)*	8.46 ± 0.21 *(6)*	18.3 ± 0.1 *(10)*	31.91 ± 0.6 (*7*)	30.96 ± 0.84 *(15)*	31.4 ± 0.63 *(11)*
HW (mg)	NTL	25.6 ± 0.5 *(9)*	52.3 ± 1.3 *(5)*	85.0 ± 1.2 *(10)*	134.2 ± 4.4 (*7*)	144.3 ± 4.9 *(9)*	128.7 ± 3.7 *(9)*
	Tg	26.1 ± 0.6 *(6)*	61.4 ± 2.2 *(6)*	105.2 ± 0.8* *(10)*	174.0 ± 3.8*** (*7*)	181.5 ± 6.6*** *(15)*	180.6 ± 4.5*** *(11)*
LVW (mg)	NTL	ND	ND	60.5 ± 1.0 *(10)*	100.1 ± 3.1 (*7*)	105.4 ± 3 *(9)*	ND
	Tg	ND	ND	75.3 ± 0.7** *(10)*	126.4 ± 3.9*** (*7*)	138.5 ± 4.3*** *(15)*	ND
TL (mm)	NTL	ND	ND	ND	17.9 ± 0.05 (*7*)	17.7 ± 0.1 *(9)*	ND
	Tg	ND	ND	ND	18.0 ± 0.08 (*7*)	17.7 ± 0.1 *(7)*	ND
LVW/TL (mg/mm)	NTL	ND	ND	ND	5.6 ± 0.2 (*7*)	6.1 ± 0.2 *(9*)	ND
	Tg	ND	ND	ND	7.0 ± 0.2*** (*7*)	7.5 ± 0.3*** *(7)*	ND

P10 and P18 mice included males and females; P ≥ 35 mice were male. Data are presented as mean ± SEM. Two-way ANOVA with Tukey’s multiple comparisons test: *p < 0.05; **p < 0.01; ***p < 0.001 NTL vs Tg. BW, body weight; HW, heart weight; LVW, left ventricular weight; TL, tibia length; LV, left ventricle; ND, not determined.

**Table 2:** NTL and dn-c-kit-Tg cardiac function during postnatal and adult development.

Parameter	Genotype	P10 *(n)*	P35 *(n)*	P196 *(n)*	P365 *(n)*
HR (bpm)	NTL	440 ± 7 *(10)*	522 ± 32 *(6)*	460 ± 17 *(9)*	513 ± 21 *(9)*
	Tg	433 ± 12 *(8)*	465 ± 15 *(8)*	460 ± 14 *(10)*	548 ± 22 *(11)*
LV internal chamber radius (*r*; mm)	NTL	1.02 ± 0.04 *(10)*	1.89 ± 0.05 *(6)*	1.74 ± 0.05 *(9)*	1.73 ± 0.05 *(9)*
	Tg	0.93 ± 0.05 *(8)*	1.71 ± 0.04 *(8)*	1.48 ± 0.07** *(10)*	1.53 ± 0.06* *(11)*
LVED wall thickness (*h*; mm)	NTL	0.58 ± 0.02 *(10)*	0.52 ± 0.02 *(6)*	1.1 ± 0.04 *(9)*	1.07 ± 0.07 *(9)*
	Tg	0.61 ± 0.04 *(8)*	0.64 ± 0.05* *(8)*	1.5 ± 0.06*** *(10)*	1.34 ± 0.10* *(11)*
Diastolic *h/r* (wall thickness/radius)	NTL	0.58 ± 0.03 *(10)*	0.28 ± 0.01 *(6)*	0.6 ± 0.03 *(9)*	0.62 ± 0.05 *(9)*
	Tg	0.68 ± 0.07 *(8)*	0.38 ± 0.03 *(8)*	1.0 ± 0.05*** *(10)*	0.92 ± 0.11** *(11)*
LVEDV (μL)	NTL	13 ± 1 *(10)*	61 ± 4 *(6)*	51 ± 4 *(9)*	50 ± 3 *(9)*
	Tg	11 ± 1 *(8)*	48 ± 3 *(8)*	35 ± 4** *(10)*	37 ± 3* *(11)*
LVESV (μL)	NTL	3 ± 0.4 *(10)*	25 ± 2 *(6)*	14 ± 1 *(9)*	15.1 ± 1.8 *(9)*
	Tg	2 ± 0.5 *(8)*	16 ± 1** *(8)*	5 ± 1*** *(15)*	5.6 ± 0.7*** *(11)*
SV (μL)	NTL	10 ± 1 *(10)*	37 ± 3 *(6)*	36 ± 2 *(9)*	35.1 ± 2 *(9)*
	Tg	9 ± 1 *(8)*	33 ± 2 *(8)*	32 ± 3 *(15)*	31.8 ± 3 *(11)*
CO (mL/min)	NTL	5 ± 0.5 *(10)*	19 ± 1 *(6)*	17 ± 1 *(9)*	20 ± 1 *(9)*
	Tg	4 ± 0.5 *(8)*	15 ± 1 *(7)*	15 ± 1 *(15)*	17 ± 2 *(11)*
LVESP (mmHg)	NTL	ND	ND	107 ± 5 *(8)*	ND
	Tg	ND	ND	112 ± 4 *(15)*	ND
LVES wall stress (mm Hg)	NTL	ND	ND	48 ± 5 *(8)*	ND
	Tg	ND	ND	17 ± 2*** *(15)*	ND
LVED wall stress (mm Hg)	NTL	ND	ND	14 ± 2 *(8)*	ND
	Tg	ND	ND	9 ± 1* *(15)*	ND
%EF	NTL	77.69 ± 1.94 *(10)*	59.87 ± 2.6 (6)	66.7 ± 2.00 *(9)*	70.5 ± 2.1 *(9)*
	Tg	78.38 ± 3.38 *(8)*	67.49 ± 1.94 (8)	79.6 ± 3.1*** *(10)*	85.3 ± 1.2*** *(11)*
Triple product [HR × dP/dt_max_ × LVES wall stress] at level of mid-papillary muscle	NTL	ND	ND	2.2 × 10^8^ ± 3.9 × 10^7^ *(8)*	ND
	Tg	ND	ND	9.2 × 10^7^ ± 1.4 × 10^7^* *(15)*	ND

P10 and P18 mice included males and females; P ≥ 35 mice were male. Data are presented as mean ± SEM. Two-way ANOVA with Tukey’s multiple comparisons test for all except LVES wall stress, LVED wall stress and Triple product where Student’s *t*-test was applied: *p < 0.05; **p < 0.01; ***p < 0.001 NTL vs Tg. HR, heart rate; LV, left ventricle; LVED, left ventricular end-diastolic; LVEDV, left ventricular end-diastolic volume; LVESV, left ventricular end-systolic volume; SV, stroke volume; CO, cardiac output; LVESP, left ventricular end-systolic pressure; EF, ejection fraction; ND, not determined.

**Table 3:** Characteristics of CMs from male NTL and dn-c-kit-Tg mice (age P112-P121)

Parameter	NTL	Tg
% 1N	10 ± 1 *(4)*	12 ± 2 *(4)*
% 2N	79 ± 2 *(4)*	74 ± 2 *(4)*
% >2N	11 ± 1 *(4)*	14 ± 3 *(4)*
% 2n	96.5 ± 0.3 *(4)*	96.2 ± 0.3 *(6)*
% 4n	3.5 ± 0.6 *(4)*	3.7 ± 0.5 *(6)*
% >4n	0.05 ± 0.01 *(4)*	0.1 ± 0.07 *(6)*
%BrdU^+^/cTnT^+^ CMs	0.013 ± 0.01 *(2)*	0.301 ± 0.07** *(2)*
%H3P^+^/cTnT^+^ CMs	0 *(2)*	0.017 ± 0.008 *(2)*
%AurB^+^/cTnT^+^ CMs	0 *(2)*	0 *(2)*
TUNEL staining	0 *(2)*	0 *(2)*
CM area, μm^2^	4898 ± 52 *(3)*	6112 ± 78* *(3)*
No. of CMs/heart	2.2 × 10^6^ ± 1 × 10^5^ *(7)*	2.1 × 10^6^ ± 3 × 10^4^ *(9)*

Data are presented as mean ± SEM. N, CM nucleation state: mono (1N), bi (2N), multi-nucleated (>2N) determined from 3,699 NTL CMs and 3,193 Tg CMs from *n* = *4* mice; % n, haploid number in CM nuclei (ploidy state) determined from 42,589 NTL and 57,676 Tg CMs from *n* = *4* and *n* = *6* mice, respectively; 115,070 NTL CMs and 88,473 Tg CMs from n = 2 mice were analysed for BrdU-positivity; 85,000 NTL CMs and 86,400 Tg CMs from *n* = *2* mice were analysed for H3P-positivity; 68,000 NTL CMs and 72,000 Tg CMs from *n* = *2* mice were analysed for AurB-positivity; 1015 NTL CMs and 682 Tg CMs from *n* = *3* mice were analysed for CM area. Student’s *t*-test: **p < 0.01; ***p < 0.001.

Echocardiographic assessments showed that at P10, NTL and dn-c-kit-Tg mice had similar cardiac dimensions and function (Table 2). However, by P35, dn-c-kit-Tg mice had a ~1.2-fold thicker LV wall (p = 0.02) and by P196, a ~30% smaller LV end-diastolic chamber volume (p = 0.002) than NTL mice. Although the *h/r* ratio (wall thickness-to-chamber radius ratio) was increased by P196 in dn-c-kit-Tg ventricles relative to NTL (p = 0.0001, Table 2, Fig. 1f), a concomitant decrease in dn-c-kit-Tg LV end-systolic volume (2.8-fold, p < 0.0001) maintained the stroke volume and cardiac output (p = ns). These changes in cardiac ventricular geometry decreased LV end-systolic wall stress (by 65%, p < 0.0001) and end-diastolic wall stress (by 36%, p = 0.02) in dn-c-kit-Tg relative to NTL (Table 2). By P196, the LV ejection fraction was considerably higher in dn-c-kit-Tg than NTL hearts (average 1.2-fold, p < 0.0001; Table 2, Fig. 1g). Hence, cardiac output in dn-c-kit-Tg mice was maintained at lower wall stress, which contributed to greater energy efficiency of the dn-c-kit-Tg hearts as evidenced by a 58% reduction (p = 0.01) in the LV triple product (heart rate × dP/dt_max_ × LVES wall stress, a measure of oxygen consumption^5^, Table 2) in dn-c-kit-Tg hearts relative to NTL. Thus, consistent with other genetic models of hypertrophy^6^, transgenic overexpression of dn-c-kit in CMs leads to a thicker LV wall, a smaller LV chamber and higher ejection fraction.

### Infarct size after acute MI was equivalent in NTL and dn-c-kit-Tg mice

Clinical studies have suggested that regional wall stress predicts ventricular remodelling and function after MI^7^. We expected that baseline differences in wall stress between genotypes would complicate assessment of post-MI changes in cardiac structure and function independent of potential cardiac regeneration. For this reason, we not only compared the effect of MI in the two genotypes, but examined changes relative to sham controls of each genotype.

**Figure 2:**
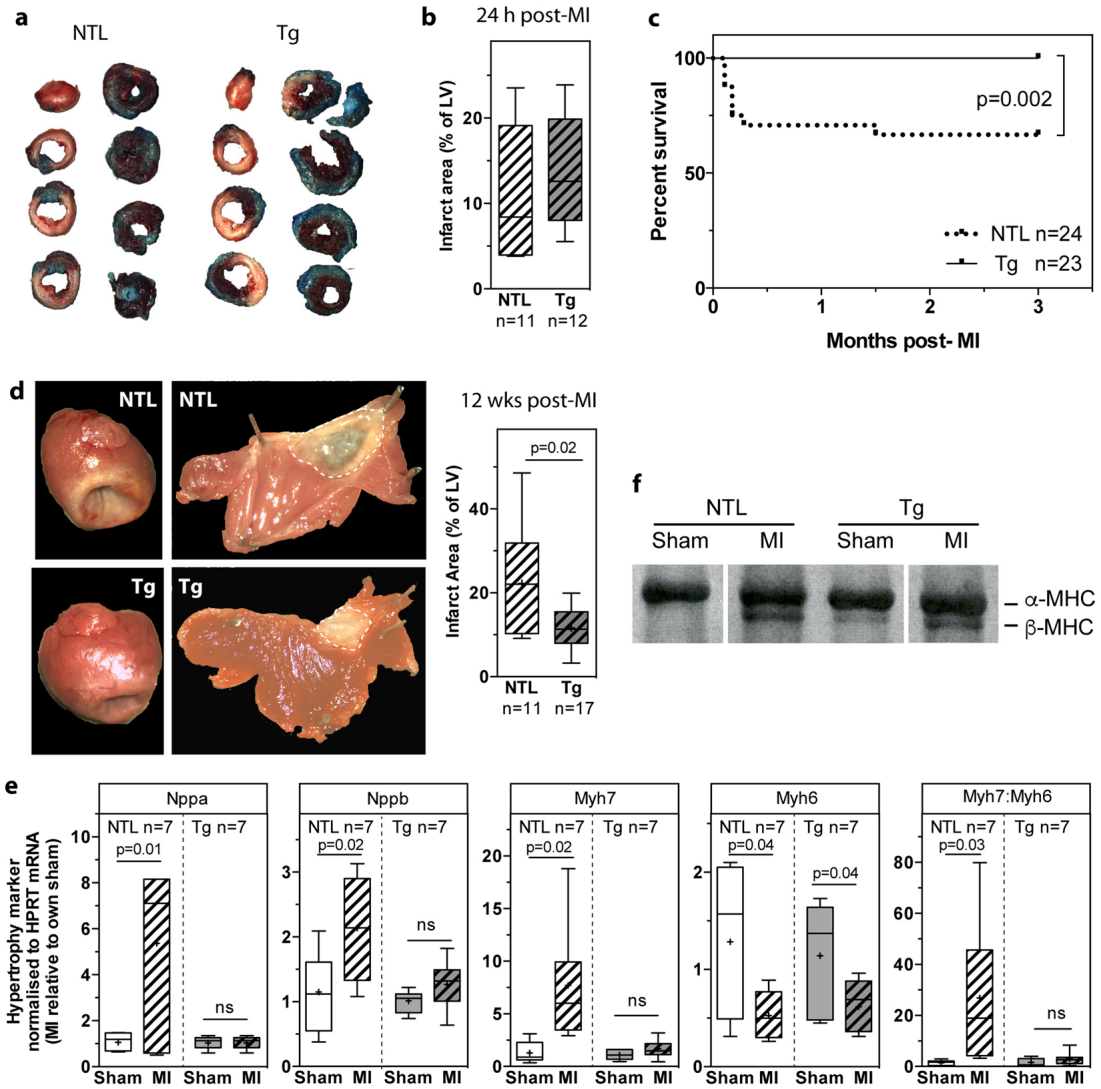
Twenty-four hours post-MI, infarct size was no different, but 12 weeks post-MI, survival was improved, infarct size was reduced and LV remodelling was absent in dn-c-kit-Tg (Tg) relative to NTL mice. (**a**) Representative photographs of Alcian-blue perfused and TTC-stained sections of NTL andTg hearts 24 h post-MI (age P113). (**b**) Twenty-four hours post-MI, there was no significant difference (analysed by Students *t*-test) between NTL (n = 11) and Tg (n = 12) hearts with respect to infarct size expressed as a percentage of the LV. (**c**) Kaplan-Meier survival curves of Tg (n = 23) and NTL (n = 24) mice after MI; analysed by log-rank test. (**d**) Left panels, representative photographs of NTL and Tg hearts 12 weeks post-MI (age P196). Middle panels, representative photographs of LVs from NTL and Tg hearts 12 weeks post-MI, with the LV cut open and pinned flat to expose the endocardial surface and the infarct area outlined by white dashed lines. Right panel, infarct size was smaller in Tg (n = 17) versus NTL (n = 11) hearts; analysed by Student’s *t*-test. (**e**) Relative to their respective shams 12 weeks post-MI, abundance of fetal gene mRNAs (*Nppa* (ANP), *Nppb* (BNP), *Myh*7 (β-MHC) and the *Myh7:Myh6* (α-MHC) ratio were higher in NTL (n = 7) but remained unchanged in Tg (n = 7) hearts, whereas abundance of *Myh6* mRNA decreased in both (age P196); differences analysed by Student’s *t*-test. (**f**) Representative silver-stained 6% SDS-polyacrylamide gel of size-fractionated heart homogenates that has been cropped (full-length gel in Supplementary Fig. S4) to show the presence of only α-MHC in NTL sham heart, and both α- and β-MHC isoforms in NTL hearts post-MI and in Tg sham and post-MI hearts (age P196).

We first assessed infarct size after acute permanent coronary artery ligation in NTL and dn-c-kit-Tg hearts. Twenty-four hours post-MI, the LV area at risk was delineated by perfusion with Alcian Blue. This was followed by TTC staining (Fig. 2a) to quantify the infarcted (or non-viable) tissue area as a percentage of the LV. We found no significant difference between genotypes with respect to LV area at risk as a percentage of the LV (NTL: 20 ± 3, n = 11 vs dn-c-kit-Tg: 22 ± 2, n = 12; p = ns), infarct size/area at risk (NTL: 59 ± 8, n = 11 vs dn-c-kit-Tg: 63 ± 3, n = 12; p = ns) or LV infarct area as a percentage of the LV (Fig. 2b), indicating that infarct size after acute MI was equivalent between NTL and dn-c-kit-Tg hearts.

### Survival was improved, and infarct size was reduced, but CM cell cycle entry was only modestly increased in dn-c-kit-Tg relative to NTL mice after chronic MI

Next, we addressed the long-term impact of MI on cardiac structure and function. NTL and dn-c-kit-Tg mice were subjected to MI or sham surgery and followed for 12 weeks post-surgery. We found no fatalities after MI in dn-c-kit-Tg mice, but ~30% of NTL mice died (Fig. 2c). Sham procedures did not result in deaths. NTL deaths mainly resulted from LV rupture within 3–7 days post-MI. At 12 weeks post-MI, infarct sizes in dn-c-kit-Tg hearts were ~50% of NTL sizes (p = 0.02) (Fig. 2d).

**Figure 3:**
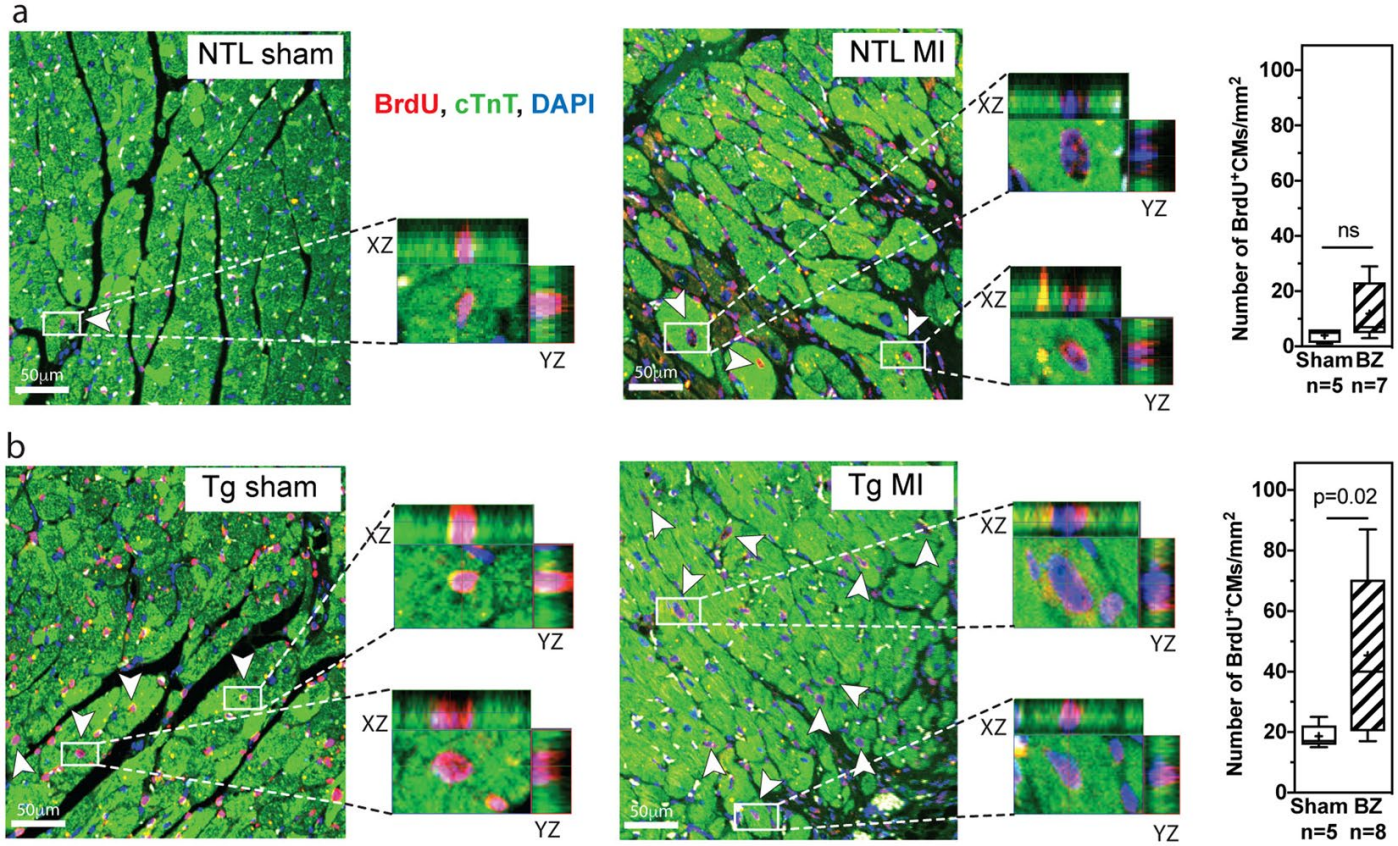
Cell cycle entry was modestly increased in dn-c-kit-Tg (Tg) but not in NTL mice post-MI. (**a**,**b**) Representative images of the border zone (BZ) of LV sections from 12 week post-sham or post-MI (age P196) NTL (**a**) or Tg (**b**) hearts co-stained for α-MHC (green), BrdU (red) and nuclei (DAPI, blue). Different focal planes (Z stacks) in the BZ in MI samples (or equivalent area in shams) were examined by confocal microscopy to identify all CMs that had cumulatively incorporated BrdU (white arrowheads). Enlarged insets are examples of XY and XZ reconstruction planes for randomly chosen α-MHC^+^/BrdU^+^ CMs. Right panels, quantitation of five representative fields, x40 objective, in the infarct BZ (or equivalent area in shams) showing the number of BrdU^+^ CMs was greater in the BZ of Tg hearts (**b**) but not NTL (**a**) hearts 12 weeks post-MI relative to sham controls; differences analysed by Student’s *t*-test. Scale bar: 50 μm.

To assess CM cell cycle entry post-MI, BrdU was infused continuously for 12 weeks, starting immediately after surgery, using implanted osmotic mini-pumps. Cumulative BrdU incorporation into CMs over three months (BrdU^+^/cTnT^+^ CMs) remained unchanged in NTL mice post-MI (p = ns) but increased by ~two-fold in the border zone relative to sham in dn-c-kit-Tg mice (p = 0.02) (Fig. 3). However, given the low percentage of CMs that progressed through mitosis and cytokinesis in adult hearts after nine days of BrdU exposure (Fig. 1c, Table 3), and the observation that this did not lead to changes in nuclear ploidy, nucleation state, or the number of CMs in the heart (Table 3), it is unlikely that the doubling of the number of CMs that were observed to have entered the cell cycle after three months of continuous BrdU exposure could effect a level of CM replacement that would be sufficient to reduce infarct sizes in dn-c-kit-Tg hearts by ~50% relative to NTL (Fig. 2d). Staining for neither H3P nor AurB was performed at this chronic time point. There was no evidence of apoptosis three months post-MI in NTL or dn-c-kit-Tg hearts by TUNEL staining (Supplementary Fig. S3, Table 5).

### LV remodelling and infarct expansion was minimal in dn-c-kit-Tg relative to NTL mice after chronic MI

LV remodelling is a consequence of MI. At 12 weeks post-MI, the LV weight-to-tibial length (LVW/TL) ratio, an index of hypertrophy, increased by 1.3-fold in NTL mice relative to sham controls (Table 4, p = 0.002). In contrast, the LVW/TL ratio remained unchanged in dn-c-kit-Tg hearts post-MI, relative to sham controls (Table 4, p = ns). Molecular markers of cardiac hypertrophy reflected this difference. In NTL hearts, abundance of *Nppa* (+5-fold, p = 0.01), *Nppb* (+2-fold, p = 0.02), *Myh*7 (+8-fold, p = 0.02) mRNAs and the *Myh*7*:Myh6* ratio (+27-fold, p = 0.03) (Fig. 2e) as well as abundance of β-MHC protein (Fig. 2f) increased post-MI. In dn-c-kit-Tg hearts, the abundance of these markers was unaltered post-MI (Fig. 2e).

**Table 4:** Cardiac function of NTL and dn-c-kit-Tg hearts three months after sham operation or myocardial infarction (age P196).

Parameter	NTL sham *(n)*				NTL MI *(n)*				Tg sham *(n)*				Tg MI *(n)*			
*Morphometry*																
BW (g)	33.2 ± 0.9 *(9)*				33.4 ± 0.7 *(12)*				32.5 ± 0.9 *(15)*				33.0 ± 0.7 *(17)*			
LV (mg)	105.4 ± 3 *(9)*				137.4 ± 6*** *(9)*				138.5 ± 4^†††^ *(14)*				145.9 ± 4 *(17)*			
LV/TL (mg/mm)	6.1 ± 0.2 *(8)*				7.8 ± 0.4** *(11)*				7.5 ± 0.3^†^ *(7)*				8.1 ± 0.3 *(12)*			
RV (mg)	26 ± 2 *(9)*				27 ± 1 *(12)*				32 ± 1 (14)				31 ± 1 *(17)*			
Atria weight (mg)	9 ± 1 *(5)*				12 ± 2 *(10)*				9 ± 1 *(5)*				11 ± 1 *(10)*			
Lung weight (mg)	163.9 ± 10 *(9)*				167.1 ± 5 *(12)*				166.8 ± 2 *(15)*				169.1 ± 5 *(17)*			
*Echocardiography*																
HR (bpm)	460 ± 17 *(9)*				465 ± 23 *(9)*				460 ± 14 *(15)*				459 ± 18 *(16)*			
LVEDV (μl)	50 ± 3 *(9)*				127 ± 25*** *(9)*				37 ± 3 *(15)*				61 ± 5^†††^ *(16)*			
LVESV (μL)	14 ± 1 *(9)*				86 ± 21*** *(9)*				5 ± 1 *(15)*				26 ± 4^†††^ *(16)*			
SV (μL)	36 ± 2 *(9)*				41 ± 5 *(9)*				32 ± 3 *(15)*				35 ± 2 *(16)*			
CO (mL/min)	17 ± 1 *(9)*				19 ± 2 *(9)*				15 ± 1 *(15)*				16 ± 1 *(16)*			
EF (%)	71.8 ± 1 *(9)*				38 ± 5*** *(9)*				87 ± 2^†^ *(15)*				61 ± 4***^†††^ *(16)*			
	mid-pap	1 mm	2 mm	3 mm	mid-pap	1 mm	2 mm	3 mm	mid-pap	1 mm	2 mm	3 mm	mid-pap	1 mm	2 mm	3 mm
LVED wall thickness, *h* (mm)	1.1 ± 0.04 *(9)*	1.0 ± 0.03 *(9)*	0.9 ± 0.03 *(9)*	0.8 ± 0.05 *(9)*	1.2 ± 0.03 *(9)*	1.1 ± 0.05 *(9)*	1.0 ± 0.05 *(9)*	0.8 ± 0.05 *(9)*	1.5 ± 0.05^†††^ *(15)*	1.3 ± 0.04^††^ *(15)*	1.2 ± 0.06^††^ *(15)*	1.0 ± 0.05^††^ *(15)*	1.4 ± 0.04 *(16)*	1.3 ± 0.05 *(16)*	1.1 ± 0.06 *(16)*	0.9 ± 0.08 *(16)*
LV internal chamber radius, *r* (mm)	1.8 ± 0.04 *(9)*	1.7 ± *0*.*05* (9)	1.4 ± 0.07 *(9)*	1.1 ± 0.06 *(9)*	2.3 ± 0.1* *(9)*	2.3 ± 0.2** *(9)*	2.2 ± 0.2*** *(9)*	2.3 ± 0.2*** *(9)*	1.5 ± 0.06 *(15)*	1.4 ± 0.07 *(15)*	1.3 ± 0.06 *(15)*	1.1 ± 0.08 *(15)*	1.8 ± 0.05^††^ *(16)*	1.7 ± 0.05^††^ *(16)*	1.6 ± 0.06^†††^ *(16)*	1.5 ± 0.1*^†††^ *(16)*
LV epicardial radius (*h* + *r)* at diastole	3 ± 0.06 *(9)*	2.8 ± 0.06 *(9)*	2.4 ± 0.08 *(9)*	1.9 ± 0.09 *(9)*	3.5 ± 0.1** *(9)*	3.4 ± 0.2** *(9)*	3.2 ± 0.2*** *(9)*	3.1 ± 0.2*** *(9)*	3 ± 0.05 *(15)*	2.8 ± 0.06 *(15)*	2.5 ± 0.08 *(15)*	2.1 ± 0.09 *(15)*	3.2 ± 0.06 *(16)*	3 ± 0.06 *(16)*	2.7 ± 0.07^††^ *(16)*	2.4 ± 0.1^†††^ *(16)*
Tissue area, SAX at diastole (mm^2^)	16.5 ± 0.8 *(9)*	14.7 ± 0.6 *(9)*	11.3 ± 0.6 *(9)*	7.3 ± 0.7 *(9)*	22.6 ± 1.2** *(9)*	19.6 ± 1.9* *(9)*	17.0 ± 1.9** *(9)*	13.7 ± 1.5** *(9)*	20.4 ± 0. 7^†^ *(15)*	17.4 ± 0.7 *(15)*	14.4 ± 1 *(15)*	10.4 ± 0.8 *(15)*	21.7 ± 0.8 *(16)*	19.0 ± 0.9 *(16)*	15.1 ± 1 *(16)*	10.8 ± 1.1*(16)*
% Fractional Area Change	68 ± 2 *(9)*	69 ± 2 *(9)*	61 ± 3 *(9)*	75 ± 4 *(9)*	44 ± 7* *(9)*	34 ± 5*** *(9)*	32 ± 6** *(9)*	23 ± 7*** *(9)*	89 ± 2^†^ *(15)*	84 ± 3 *(15)*	85 ± 5^†^ *(15)*	79 ± 6 *(15)*	75 ± 2^†††^ *(16)*	60 ± 3**^††^ *(16)*	45 ± 6*** *(16)*	41 ± 8*** *(16)*
*Micromanometry*																
HR (bpm)	469 ± 6 *(8)*				463 ± 6 *(11)*				472 ± 5 *(15)*				470 ± 3 *(16)*			
LVEDP (mm Hg)	7 ± 0.5 *(8)*				5 ± 0.6 *(11)*				9 ± 1 *(15)*				9 ± 0.6^†^ *(16)*			
LVESP (mm Hg)	107 ± 5 *(8)*				99 ± 4 *(11)*				112 ± 4 *(15)*				109 ± 3 *(16)*			
PRSW slope (single beat; mmHg/s)	218.7 ± 15.1 *(9)*				115.7 ± 20*** *(8)*				290.6 ± 7.3^††^ *(15)*				199.5 ± 16.7***^††^ *(16)*			

Data are presented as mean ± SEM. n, number of animals. Ordinary one-way or two-way ANOVA with Tukey’s multiple comparisons tests: *p < 0.05, **p < 0.01, ***p < 0.001 MI mice vs their respective sham-operated controls and ^†^p < 0.05, ^††^p < 0.01, ^†††^p < 0.001, NTL vs Tg mice BW, body weight; LV, left ventricle; TL, tibia length; RV, right ventricle; HR, heart rate; LVEDV, left ventricular end-diastolic volume; LVESV, left ventricular end systolic volume; SV, stroke volume; CO, cardiac output; EF, ejection fraction; SAX, short axis view; LVEDP, left ventricular end-diastolic pressure; LVESP, left ventricular end-systolic pressure; PRSW, preload recruitable stroke work.

The size of the MIs produced by the coronary artery ligation procedure was not sufficiently large to induce heart failure in NTL or dn-c-kit-Tg mice post-MI. This is reflected by the findings that in both genotypes right ventricular weights and lung weights were not significantly increased relative to their respective shams (Table 4), nor were levels of fibrosis in regions remote to the infarct (Table 5). While loss of c-kit function in endothelial c-kit+ stem cells causes defective myocardial angiogenesis^2^, we found no significant difference between NTL and dn-c-kit-Tg hearts in capillary density, CM-to-capillary ratio or mean inter-capillary distance in the area remote from the infarct (Table 5). Thus, CM-restricted overexpression of dn-c-kit did not impact coronary architecture.

**Table 5:** Histology of NTL and dn-c-kit-Tg hearts three months after sham operation or myocardial infarction (age P196).

Parameter	NTL sham *(n)*	NTL MI *(n)*	Tg sham *(n)*	Tg MI *(n)*
Percent fibrosis	0.9 ± 0.01 *(5)*	1.1 ± 0.2 *(7)*	0.8 ± 0.1 *(5)*	1.2 ± 0.3 *(9)*
Capillary density in remote zone (capillaries/mm^2^)	3329 ± 332 *(6)*	3340 ± 445 *(8)*	2967 ± 230 *(6)*	3673 ± 577 *(8)*
Capillary density/CM	1.54 ± 0.09 *(6)*	1.47 ± 0.1 *(8)*	1.49 ± 0.06 *(6)*	1.54 ± 0.07 *(8)*
Mean intercapillary distance (μm)	0.03 ± 0.002 *(6)*	0.03 ± 0.001 *(8)*	0.03 ± 0.001 *(6)*	0.03 ± 0.002 *(8)*
TUNEL staining	0 *(3)*	0 *(3)*	0 *(3)*	0 *(3)*

Data are presented as mean ± SEM. n, number of animals. Ordinary one-way ANOVA with Tukey’s multiple comparisons tests: *p < 0.05, **p < 0.01, ***p < 0.001 MI mice vs their respective sham-operated controls and ^†^p < 0.05, ^††^p < 0.01, ^†††^p < 0.001, NTL vs Tg mice^.^ CM, cardiomyocyte.

LV chamber dilatation, a feature of post-MI cardiac remodelling is the principle cause of infarct expansion^7^. LV chamber dilatation was observed in both genotypes, but it was more marked in NTL hearts post-MI. This was evident from changes in i) LV internal chamber radius [NTL hearts: +30 (p = 0.03), +35 (p = 0.005), +57 (p < 0.0001), +109% (p < 0.0001) post-MI compared to sham controls at the mid papillary level and 1, 2 and 3 mm distal, respectively, versus dn-c-kit-Tg hearts: +36% (p = 0.01) post-MI compared to sham controls at 3 mm distal to the mid-papillary level], ii) LV end-diastolic volume (LVEDV) [NTL hearts: +2.54-fold post-MI compared to their sham controls, p = 0.0001, versus dn-c-kit-Tg hearts: no significant difference post-MI compared to their sham controls], and iii) tissue area in short-axis (SAX) view [NTL hearts: +1.3 (p = 0.002), +1.3 (p = 0.022), +1.5 (p = 0.005), +1.9 (p = 0.002) post-MI compared to their sham controls versus dn-c-kit-Tg hearts: no significant difference post-MI compared to their sham controls] (Table 4). As noted above, LV infarct area, as a percentage of LV area, in dn-c-kit-Tg hearts was about half that of NTL mice (Fig. 2d), and was no greater 12 weeks post-MI than 24 h post-MI, whereas in NTL mice, infarct area 12 weeks post-MI was ~two-fold greater than 24 h post-MI. This is likely due to greater infarct expansion and LV wall thinning of infarcts in NTL than in dn-c-kit-Tg hearts. Thus, our findings indicate a lack of post-MI infarct expansion in dn-c-kit-Tg hearts.

### Cardiac function was preserved in dn-c-kit-Tg mice relative to NTL mice after chronic MI

**Figure 4:**
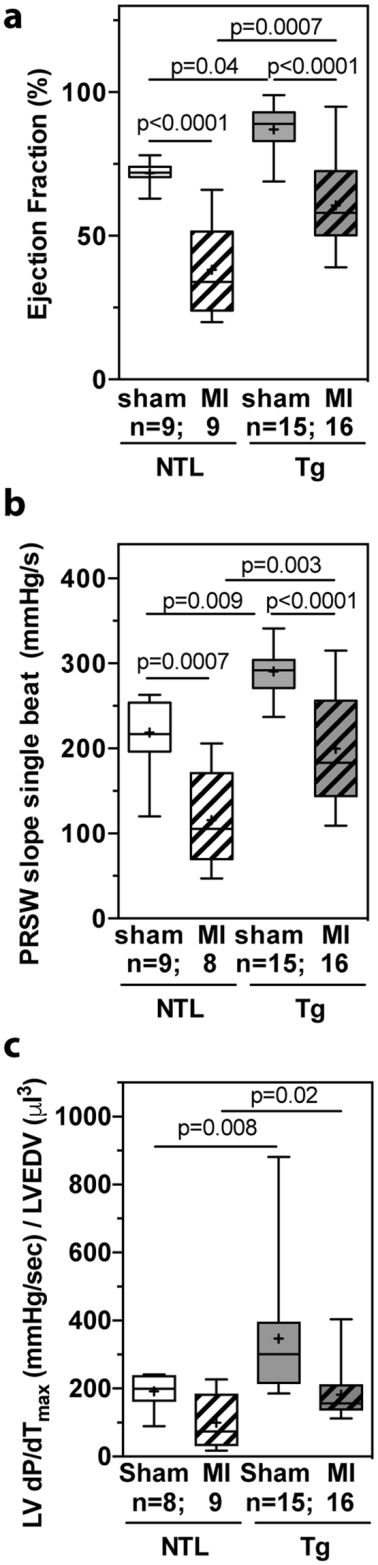
Cardiac contractile function 12 weeks post-MI was better preserved in dn-c-kit-Tg (Tg) relative to NTL hearts. (**a**) EF was greater in P196 Tg sham (n = 15) and post-MI hearts (n = 16) relative to NTL controls (n = 9), and post-MI loss of ejection fraction was less in Tg than in NTL hearts, relative to respective sham controls; differences analysed by ordinary one-way ANOVA with Tukey’s multiple comparison test. (**b**) Single beat PRSW slopes indicate contractility was greater in P196 Tg sham (n = 15) and post-MI (n = 16) hearts relative to NTL (n = 8–9) hearts, and post-MI loss of contractility of Tg hearts was less than that of NTL hearts relative to their respective sham controls; differences analysed by ordinary one-way ANOVA with Tukey’s multiple comparison test. (**c**) dP/dt_max_ normalised to LVEDV was greater in P196 sham Tg (n = 15) relative to NTL (n = 8) hearts and in post-MI Tg (n = 16) relative to NTL (n = 9) hearts; differences analysed by Student’s *t*-test.

We also assessed cardiac functional consequences of MI in NTL and dn-c-kit-Tg mice. Compared to their respective shams, the LV ejection fraction (LVEF) post-MI (Fig. 4a, Table 4) decreased by almost half in NTL mice from 72% to 38% (p < 0.0001), but only by a third in dn-c-kit-Tg hearts from 87% to 61% (p < 0.0001). Differences between the genotypes in regional contractile function, indexed by the fractional area change between end-diastole and end-systole, were less pronounced. Thus, compared with their respective sham controls at the mid papillary level and 1, 2 and 3 mm distal, fractional area change decreased by −35 (p = 0.03), −51 (p = 0.0003), −48 (p = 0.004) and −69% (p < 0.0001), respectively, in NTL hearts post-MI versus 0 (p = ns), −29 (p = 0.0024), −47 (p < 0.0001), −48% (p < 0.0001), respectively, in dn-c-kit-Tg hearts (Table 4). LV stroke volume and cardiac output were similar in NTL sham and dn-c-kit-Tg sham mice, and remained unaltered post-MI (Table 4, p = ns). The single beat preload recruitable stroke work (PRSW) slope, a load-independent index of cardiac performance^8^, indicated that contractility of dn-c-kit-Tg sham hearts was 1.3-fold higher than in NTL sham hearts before MI (p = 0.009; Fig. 4b, Table 4). Post-MI, the PRSW slope in NTL hearts was reduced to almost half of that in sham controls (p = 0.007; Fig. 4b, Table 4) whereas in dn-c-kit-Tg hearts the reduction in PRSW slope was markedly less, with the post-MI PRSW slope being ~70% of that of their sham controls (p < 0.0001; Fig. 4b, Table 4). Thus, although the PRSW slope fell post-MI in both genotypes, it was better maintained in dn-c-kit-Tg hearts (−31.3%) than in NTL hearts (−47%). Better preservation of myocardial contractility in dn-c-kit-Tg mice was also evident from another load-independent index: LV dP/dt_max_ normalized to EDV^9^, which was 1.8–fold higher in dn-c-kit-Tg relative to NTL hearts, both in sham (p = 0.008) and in post-MI groups (p = 0.02, Fig. 4c).

Indices of diastolic LV function also differed between the genotypes post-MI. In NTL hearts, LV dP/dt_min_, fell post-MI (sham: −9132 ± 609 n = 8, MI: −6844 ± 699 mmHg/s n = 11; p = 0.03) and Tau, a preload-independent measure of isovolumic relaxation, increased 1.9-fold (sham: 11 ± 0.3 n = 8, MI: 21 ± 4 ms n = 11; p = 0.04). In contrast, both of these indices remained unchanged post-MI in dn-c-kit-Tg hearts (dP/dt_min_: Sham, −8795 ± 902 n = 15; MI, −7542 ± 306 mmHg/s n = 16; p = ns. Tau: Sham, 15 ± 3 n = 15; MI, 14 ± 1 ms n = 16; p = ns).

## Discussion

Primary cardiac hypertrophy is the major phenotype of clinical forms of hypertrophic cardiomyopathy that are associated with mutations in the sarcomeric genes, including myosin-binding protein C, β-myosin heavy chain and cardiac troponin T^10^. This is manifested by increased cardiomyocyte size; myofibrillar disarray; altered LV geometry, including diminished LV chamber diameter; myocardial fibrosis; enhanced expression of fetal genes encoding atrial (*Nppa*) and brain (*Nppb*) natriuretic factor, α-myosin heavy chain (*Myh6*, or in rodents, *Mhy*7) and α-skeletal actin (*Acta1*); and an altered myocardial vasculature^6,11–14^. However, even amongst patients with the same mutation, there is considerable variation in symptoms and severity with clinical manifestations ranging from benign to severe heart failure and sudden death^12,15,16^. These hallmarks of hypertrophy are also observed in mouse models of hypertrophic cardiomoyopathy^6,17^. The primacy of the genetic change as the underlying cause of CM enlargement in these mouse models is the lack of hypertension or other factors that would stimulate hypertrophy by increasing afterload. Many of these phenotypic changes were observed basally in the dn-c-kit-Tg model, including LV hypertrophy (increased LVW/TL ratio, increased LVED wall thickness) with decreased LV cavity size (decreased LV internal chamber radius) and CM hypertrophy (increased CM area) in the absence of an increase in afterload (LVSP unchanged) as well as activation of the fetal gene program (enhanced *Nppa*, *Nppb* and *Myh*7 mRNA levels). Hence, dn-c-kit-Tg mice display the hallmarks of primary concentric cardiac hypertrophy.

Although activation of the fetal gene program is generally observed in pathological forms of hypertrophy, it is of interest that in the dn-c-kit-Tg hearts, hypertrophy occurred in the absence of other markers of pathological hypertrophy, such as decreased *Myh6* expression and increased *Acta1* expression, myofibrillar disarray, increased myocardial fibrosis, altered capillary density or increased apoptosis. Moreover, because both end-systolic and end-diastolic chamber volumes were reduced commensurately in dn-c-kit-Tg hearts, stroke volume and cardiac output were maintained. The most important consequence of this altered LV geometry was that end-systolic and end-diastolic wall stresses were markedly lower in dn-c-ki-Tg hearts than in age-matched NTL hearts and, as a result, LV triple product was lower in dn-c-kit-Tg hearts. Thus, despite frank concentric hypertrophy, these features of dn-c-kit-Tg hearts provide a unique opportunity to evaluate factors that might contribute to variations in severity of hypertrophic cardiomyopathies and that might also mitigate sequelae post-MI.

After MI, infarct size increases by extension and expansion; the latter being an important risk factor for death by LV wall rupture or aneurysm formation^7^. Infarct extension is the product of apoptotic and necrotic cell death. We show here that coronary artery ligation in dn-c-kit-Tg and WT hearts produced equivalent infarct sizes at 24 hours post-MI. This finding suggests that neither cardiac cell survival nor apoptosis were significantly altered by dn-c-kit expression in CMs. Infarct expansion is mainly due to LV wall thinning and chamber dilatation, which leads to increased wall stress^7^. Analysis of 12 week post-MI hearts indicated markedly expanded infarcts in NTL but not in dn-c-kit-Tg hearts. In keeping with this, remodelling of the LV of dn-c-kit-Tg hearts was much less than that of NTLs after MI. These findings are entirely consistent with clinical studies based on autopsy findings, which indicate that infarct expansion is less marked in hearts with ventricular hypertrophy^3^, whereas increased wall stress is a stimulus for cardiac hypertrophy^18,19^ and predicts both ventricular remodelling and impaired function after MI^7^. However, it was unclear from these clinical studies if the hypertrophy observed post-mortem was pre-existing or due to remodelling of non-infarcted myocardium. The finding that infarct expansion occurs more commonly with infarcts involving the left anterior descending coronary artery distribution, a region of the LV with the greatest curvature, has been suggested to indicate that the degree to which an infarct expands may be influenced by the pre-infarct thickness of the ventricular wall^3^. In that study^3^, it was not possible to exclude the possibility that protection from infarct expansion was due to the infarcts being smaller in the hypertrophic heart. The finding that infarct size remained unchanged in dn-c-kit-Tg hearts, which have pre-existing hypertrophy with reduced LV wall stress and improved energetics, therefore, strongly supports the notion that pre-infarct hypertrophy limits infarct expansion. Moreover, lower wall stress and attenuated infarct expansion reduce the risk of LV wall rupture^7^, which likely explains the improved survival of dn-c-kit-Tg mice post-MI, with most deaths in the NTL mice occurring 3–7 days post-MI due to ventricular rupture.

While c-kit inactivation in CMs can cause CMs to retain proliferative competence, as we have shown previously using a pressure-overload model^9^, in the current study we found that MI did not trigger meaningful increases in CM cell cycle entry in the infarct border zone of dn-c-kit-Tg hearts. Both preload and afterload are increased in left ventricles post-MI^20^. But unlike increased ventricular afterload due to aortic constriction^9^, MI also reduces the CM population in the infarct zone, and the load-increase in the border zone and remote zones is modest when compared with the load imposed by aortic constriction. This is particularly the case in dn-c-kit-Tg mice because wall stress prior to MI was markedly lower and LV dilatation post-MI was much reduced, relative to NTL mice. In addition, effective cardiac regeneration requires simultaneous resorption of the post-infarct scar tissue as well as robust CM proliferation to achieve complete repair of the myocardium, as observed in the regenerating adult zebrafish heart^21^ and the neonatal murine heart^22^. Systemic and local immune responses play critical roles in scar formation and resorption^23^. In neonatal hearts the acute inflammatory response stimulates the regenerative response^24^, but in adults it accelerates infarct progression. It is possible that an aggressive post-MI inflammatory response in adulthood attenuates pro-proliferative signals caused by injury. Future studies should help in understanding the role of inflammatory mediators, which are activated early after MI injury, in the cardiac regenerative process in adults.

## Methods

### Ethics Statement

All experimental procedures were approved by the Garvan Institute/St. Vincent’s Hospital Animal Experimentation Ethics Committee (No. 10/18, 13/07) and were performed in strict accordance with the National Health and Medical Research Council (NHMRC) of Australia Guidelines on Animal Experimentation. All efforts were made to minimize suffering. All analyses were performed with the operator blinded to genotype.

### Mice

Heterozygous α-MHC/*W*^*v*^-Tg-2.1 mice (designated here as dn-c-kit-Tg), with cardiac-restricted overexpression of the dominant-negative c-kit^*Wv*^ (T660M) mutant protein, were generated on a pure C57BL/6J background as described^1^. Heterozygous dn-c-kit-Tg and NTL generated from dn-c-kit-Tg × NTL crosses were used.

### Adult CM isolation

CMs were isolated from male mice from litters of 6–8 in size. Hearts were excised and perfused as described^25^ with the following modification: perfusion buffer contained 3 mg/mL collagenase (Worthington) for 8 min at 37 °C. For downstream immunocytochemistry, area measurement and determination of nucleation status, hearts were perfused with 2 mL freshly thawed 2% PFA before disaggregation; for downstream CM counting and ploidy determination, hearts were not fixed. Hearts were gently disaggregated by teasing apart the tissue with forceps for 5 mins in a petri dish before passaging through transfer pipettes of progressively smaller diameters. Dissociated PFA-fixed cells were diluted to 15 mL with PBS and washed (2 × 5 mins, 168 *g*) to remove PFA.

### Immunocytochemistry of isolated CMs

To enable evaluation of CMs undergoing cell cycle entry (BrdU), metaphase (phospho-histone H3, H3P) or anaphase (Aurora kinase B, AurB), osmotic mini-pumps (Alzet Model 2002) were implanted subcutaneously into the flank of male mice (16 weeks old, n = 4) to deliver 5-bromo-2′-deoxyuridine (BrdU, 25 mg/mL in 50% DMSO/water to deliver 10 mg/kg/day) for nine days. Briefly, PFA-fixed CMs were cytospun onto Superfrost slides, and fixed again (2% PFA, 5 mins, RT). Slides were washed with 0.1 M glycine to quench any unreacted aldehyde. For heat-induced antigen retrieval, slides were incubated (96 °C, 5 mins) in sodium citrate buffer and allowed to cool in the buffer for 1 h. For AurB staining, endogenous peroxidases were quenched (0.3% (v/v) H_2_O_2_ in 70% (v/v) methanol in PBS, 30 min, RT, rocking). All slides were blocked and permeabilized for 1 h (for BrdU or H3P staining: 5% (v/v) normal donkey serum, 0.2% PBS-Triton X-100 (PBS-T); for AurB staining: 3% (v/v) normal donkey serum, 3% BSA (v/v), 0.1% PBS-T) before incubating overnight at 4 °C with primary mouse anti-cTnT monoclonal antibody (Abcam, ab10214, 1:600) and rat anti-BrdU monoclonal antibody (Abcam, ab 6326, 1:100), rabbit anti-H3P polyclonal antibody (Abcam, ab5176, 1:60) or rabbit anti-AurB polyclonal antibody (Abcam, ab2254, 1:50). Slides were then incubated for 1 h at RT with species-appropriate secondary donkey anti-mouse-488 (Jackson laboratory, 715-545-151, 1:500) and donkey anti-rat-RRX (Jackson laboratory, 712-295-153, 1:500 in 5% (v/v) normal donkey serum, 0.01% PBS-T), donkey anti-rabbit-RRX (for H3P, Jackson laboratory, 711-295-152, 1:500 in 5% (v/v) normal donkey serum, 0.01% PBS-T) or donkey anti-rabbit-biotin (for AurB, Jackson laboratory, 711-065-152, 1:250 in 1% (v/v) BSA in PBS). For AurB staining, slides were incubated with ABC complex (VECTASTAIN^®^ Elite^®^ ABC-HRP kit, PK-6101) for 1 h at RT, then with Tyramide-cys3 (TSA Cyanine 3 system, NEL704A001KT, 1:100) for 3 min. Nuclei were stained with 4′,6-diamidino-2-phenylindole (DAPI, Sigma Aldrich, D9542) before mounting coverslips. cTnt^+^/BrdU^+^, cTnt^+^/H3P^+^ or cTnt^+^/AurB^+^ CMs were counted under the confocal microscope (Zeiss LSM 700 Upright, MicroImaging GmbH, 07740 Jena, Germany) and representative images taken. CMs isolated from two-day old (P2) hearts were used as controls for H3P and AurB antibody staining.

### Area measurement and nucleation status of isolated CMs

PFA-fixed CMs were cytospun onto Superfrost slides, blocked with 5% (v/v) normal donkey serum (1 h), incubated with primary mouse anti-cTnT monoclonal antibody (Abcam, ab 10214, 1:600, overnight, 4 °C), then secondary antibody donkey anti-mouse-488 (Jackson laboratory, 715-545-151, 1:500, 1 h, RT). Nuclei were stained with TO-PRO^TM^-3 Iodide (642/661) (Invitrogen, T3605) before mounting coverslips. CM surface area was calculated from 100 fields (20x objective, Zeiss LSM 7 DUO, tiling function, MicroImaging GmbH, 07740 Jena, Germany) using ImageJ. CM nucleation was calculated using the cell counter plugin (https://imagej.nih.gov/ij/plugins/cell-counter.html).

### Counting and ploidy determination of isolated CMs

CMs were counted on a haemocytometer in Transfer Buffer A^25^. After counting, CMs were fixed in ice-cold 70% v/v ethanol. Nuclei were liberated by digestion with 0.2 M HCl and 1 mg/mL pepsin (37 °C, 15 min)^26^ and pelleted. Nuclei were stained with 50 μg/mL propidium iodide with 12 μg/mL RNase (22 °C, 30 min) and analysed using a Canto II flow cytometer (BD). At least 30,000 nuclei were analysed per heart. P4 CMs, which are in majority diploid, were used as ploidy control.

### TUNEL staining

Apoptosis in heart sections was detected by *In Situ* Cell Death Detection Kit, TMR red (Roche) according to manufacturer’s instructions.

### Myocardial infarction and infarct size determination

Male mice (16 weeks old) were randomised, anaesthetised (ketamine: 100 mg/kg, xylazine: 13 mg/kg, atropine: 0.5 mg/kg) by intraperitoneal injection, placed on a heating pad, intubated and ventilated (120 breaths/min). Depth of anaesthesia was monitored by respiration rate, colour of mucous membranes and skin, and toe pinch reflex. The heart was accessed by an incision of the left wall of the chest at the fourth intercostal space. Mice were subjected to myocardial infarction (MI) by permanent ligation of the left anterior descending coronary artery (8/0 prolene suture) at about 2 mm below the edge of the left auricle, or to sham operation. The chest was closed and the pneumothorax reduced. Mice were monitored continuously during recovery until the righting reflex was regained, and housed overnight half on a heating pad. Post-operative analgesia (buprenorphine, 0.075 mg/kg) was administered subcutaneously twice daily for three days. To cumulatively label cells entering S phase, an osmotic mini-pump (Alzet Model 2006) was implanted subcutaneously into the flank immediately after surgery to deliver BrdU (83 mg/mL in 50% DMSO/water; 10 mg/kg/day for 6 weeks) and replaced 6 weeks post-surgery under isoflurane (1–2%) anaesthesia, with post-operative analgesia (bupivacaine, 8 mg/kg) administered subcutaneously at the wound site for three days after surgery.

Infarct size was determined at 24 h post-MI by planimetry of infarcted tissue in 1 mm thick LV coronal slices (mouse heart slicer matrix, Zivic Instruments) following perfusion with 0.02% Alcian-blue, staining with 2,3,5-triphenyltetrazolium chloride (1% in phosphate-buffered saline) and fixation (10% formalin solution in neutral buffer) as described^27^. To measure infarct size 12 weeks post-MI, the heart was excised immediately following hemodynamic measurements, atria and right ventricle were trimmed off, the LV was cut open and pinned flat to expose the endocardial surface for determination by planimetry of LV wall scar tissue as a percentage of the total LV chamber area^28^. LV tissue was cut parasagitally into thirds so that each piece contained a portion of infarcted tissue. One piece was processed for paraffin-embedding and two pieces were snap-frozen in liquid nitrogen for quantitative RT-PCR and protein analyses.

### Echocardiography

Echocardiography was performed on isoflurane-anaesthetized mice (~1–2%) using a Vevo 770 cardiac ultrasound system (VisualSonics) on a heating pad. After shaving the chest and washing with 70% ethanol, ultrasound gel was applied to the chest wall and B-mode images were taken of LV long axis (LAX) followed by four short axis (SAX) views at the mid-papillary level, and then at the levels 1, 2 and 3 mm toward the apex. LV length was measured from the apical dimple to the base of the aortic valve leaflets from the LAX images at end-diastole and end-systole. The areas boarded by the endo- and epicardium were determined by planimetry of all four SAX images at end-diastole and end-systole, and from this a modified Bullet formula was applied to derive LVED and LVES volumes (LV_vol_ = L × (mean endocardial area) × 5/6). In addition, tissue cross-sectional area (CSA) and fractional area change (FAC) for each SAX image were calculated. Tissue CSA was calculated as the difference between the epicardial CSA and the endocardial CSA at end-diastole. FAC was calculated as the difference between the end-diastolic and end-systolic endocardial CSA divided by the end-diastolic endocardial CSA, and expressed as a percentage. Mean chamber radius (r = √(endocardial area/π) and wall thickness (h = √(epicardial area/π) - r) were calculated from the end-diastolic CSA data. The acquisition of images and evaluation of data were performed by an operator who was blinded to the genotype and treatment of the mice.

### Hemodynamic Measurements

Hemodynamic measurements were performed on isoflurane-anaesthetized (~1–2%) mice using a 1.2 F micromanometer transducer tipped catheter (Scisense Inc, Toronto, CA) that was passed via the right carotid artery into the aorta (for measurement of central arterial pressure) and then advanced into the LV of the heart. LV pressure and heart rate were monitored until stable recordings were obtained at a heart rate of ~500 bpm. Immediately following hemodynamic measurements, anaesthetized mice were euthanised by cervical dislocation and hearts were collected for analysis.

### Quantitative RT-PCR

RNA isolated from a third of a snap-frozen LV was subjected to RT-qPCR as described^29^. Using the 2^−ΔΔCT^ method, relative expression (fold change) of c-kit (*Kit*, TaqMan assay ID: Mm00445212_m1, amplicon length 71 bp spanning exons 7–8, context sequence: 5′-TTTACGTGAACACAAAACCAGAAAT-3′ with assay location at 1306 of NM_001122733.1), α-skeletal actin (*Acta1*, TaqMan assay ID: Mm00808218_g1, amplicon length 134 bp spanning exons 4–5, context sequence: 5′-CTTCCGGCCGTACCACCGGCATCGT-3′ with assay location at 635 of NM_001272041.1), α-myosin heavy chain (*Myh6*, TaqMan assay ID: Mm00440359_m1, amplicon length 67 bp spanning exons 20–21, context sequence: 5′-GGAACGCAGGGATGCCCTGCTGGTT-3′ with assay location at 2635 of NM_001164171.1), β-myosin heavy chain (*Myh*7, TaqMan assay ID: Mm00600555_m1, amplicon length 102 bp spanning exons 40–41, context sequence: 5′-CGGGACATTGGTGCCAAGGGCCTGA-3′ with assay location at 5926 of NM_080728.2), atrial natriuretic peptide (*Nppa*, TaqMan assay ID: Mm01255747_g1, amplicon length 85 bp spanning exons 1–2, context sequence: 5′-GATGGATTTCAAGAACCTGCTAGAC-3′ with assay location at 209 of NM_008725.2) and brain natriuretic peptide (*Nppb*, TaqMan assay ID: Mm01255770_g1, amplicon length 68 bp spanning exons 2–3, context sequence: 5′-GTTTGGGCTGTAACGCACTGAAGTT-3′ with assay location at 534 of NM_008726.4) was normalized to hypoxanthine-guanosine phosphoribosyltransferase (*Hprt*, TaqMan assay ID: Mm01545399_m1, amplicon length 81 bp spanning exons 2–3, context sequence: 5′-GGACTGATTATGGACAGGACTGAAA-3′ with assay location at 276 of NM_013556.2) as the most suitable reference gene (expression level unaffected by the experimental treatment) and relative to the NTL sham-operated group as the calibrator. A standard curve using cDNAs diluted to 1:10, 1:50, 1:100, and 1:1,000 (slopes between −3.1 and −3.5, y intercepts 18–24) gave similar PCR amplification efficiencies for the reference and target genes (90–110%), thereby validating the use of the 2^−ΔΔCT^ method for each gene examined. A no-template control reaction was included for each gene examined.

### MHC protein identification

Protein from a third of a snap-frozen LV was enriched for myofibrillar proteins^30^ and subjected to SDS-PAGE and silver staining.

### Immunohistochemistry

To evaluate CM cell cycle entry, a third of a LV was fixed in 2% paraformaldehyde for 4 h, stored in 70% ethanol and then paraffin-embedded. Sections (5 μm) were deparaffinised in xylene, rehydrated in a series of graded alcohols, stained with primary mouse anti-MHC[3–48] antibody (Abcam, ab 11575, 1:200) and BrdU rat monoclonal antibody (Abcam, ab 6326, 1:100) in 5% goat serum (overnight, 4 °C), and visualised with goat anti-mouse 488 (green) (Invitrogen, A-11029) and goat anti-rat 594 (red) antibody (Invitrogen, A-11007). Nuclei were stained with DAPI (Sigma Aldrich, D9542) before mounting coverslips. Images (five representative fields in the infarct border, or equivalent region in the sham, per section, 20x objective) were acquired (20x objective, Zeiss LSM 700 Upright, MicroImaging GmbH, 07740 Jena, Germany). Different focal planes (Z stacks) were examined to ensure nuclei of interest were located in CMs.

For capillary density determination, sections (5 μm) were stained for endothelial cells with fluorescein-labelled *Griffonia (Bandeiraea) simplicifolia* isolectin B4 (FL-1201, Vector laboratories, 1:100, 1 h, RT), cell membranes with rabbit anti-laminin polyclonal antibody (red) (Abcam, 11575, 1:100) and CMs with mouse anti-MHC monoclonal antibody (red) (Abcam, ab-15, 1:200). Images (whole section) were acquired (Leica DM 6000 power mosaic, 40x objective). Three to six representative fields remote to the infarct region, with capillaries and CMs in cross-section, were evaluated for total field size and the number of CMs and capillaries within the field were counted.

### Fibrosis

Paraffin sections (5 μm) were stained (0.1% picrosirius red (Picric acid-Sigma, P6744, Direct Red 80- Sigma, 365548) with 0.1% fast green FCF (Sigma, F7258), 1 h. Images (three representative fields remote to the infarct region, or equivalent region in the sham, per section) acquired under polarized light (20x objective, Leica DM 6000 power mosaic) were analysed for fibrosis by semi-automated quantitation (ImageJ v 1.46r), with colour transformation to binary colours and threshold adjustment as described^31^.

### Statistics

Data in figures are presented 1) as box (extending from the 25th to 75th percentiles) and whisker (extending from max to min) plots showing the mean (represented by “+”) and the median (represented by the line in the middle of the box), or 2) scatter plot showing mean ± 1 S.E. of the mean (SEM). Data in tables are presented as mean ± 1SEM. Statistical differences of continuous variables were determined by unpaired, two-tailed Student’s *t* test for orthogonal comparisons, ANOVA plus Tukey’s multiple comparison tests or log-rank test, where appropriate. If variances in Student’s *t* tests were unequal, Welch’s correction was applied. GraphPad Prism software was used for statistical analyses. p < 0.05 was considered significant.

### Data availability

The data that support the findings of this study are included in this published article and its supplementary information files.

## Electronic supplementary material

Supplementary Figures 1-4
