# Supplementary material for: Cardiac hypertrophy limits infarct expansion after myocardial infarction in mice
Source: Sci Rep. 2018 Apr 17;8:6114. doi: 10.1038/s41598-018-24525-6 (PMC5904135; doi:10.1038/s41598-018-24525-6)
Supplement: Supplementary file 1 — Supplementary Figures 1-4 [file 41598_2018_24525_MOESM1_ESM.pdf]

Cardiac hypertrophy limits infarct expansion after myocardial infarction in mice

Siiri E. Iismaa[a], Ming Li, Scott Kesteven, Jianxin Wu, Andrea Y. Chan, Sara R. Holman, John W. Calvert, Ahtesham ul Haq, Amy M. Nicks, Nawazish Naqvi, Ahsan Husain, Michael P. Feneley and Robert M. Graham

Supplementary Information

Supplementary Figure S1: Ploidy of CMs isolated from NTL or dn-c-kit-Tg (Tg) hearts is equivalent.

Supplementary Figure S2: No evidence of myofibrillar disarray or CM apoptosis in P112 NTL or dn-c-kit-Tg (Tg) hearts.

Supplementary Figure S3: No evidence of CM apoptosis in NTL or dn-c-kit-Tg (Tg) hearts 12 weeks post-MI.

Supplementary Figure S4: Full-length gel from Figure 2f.

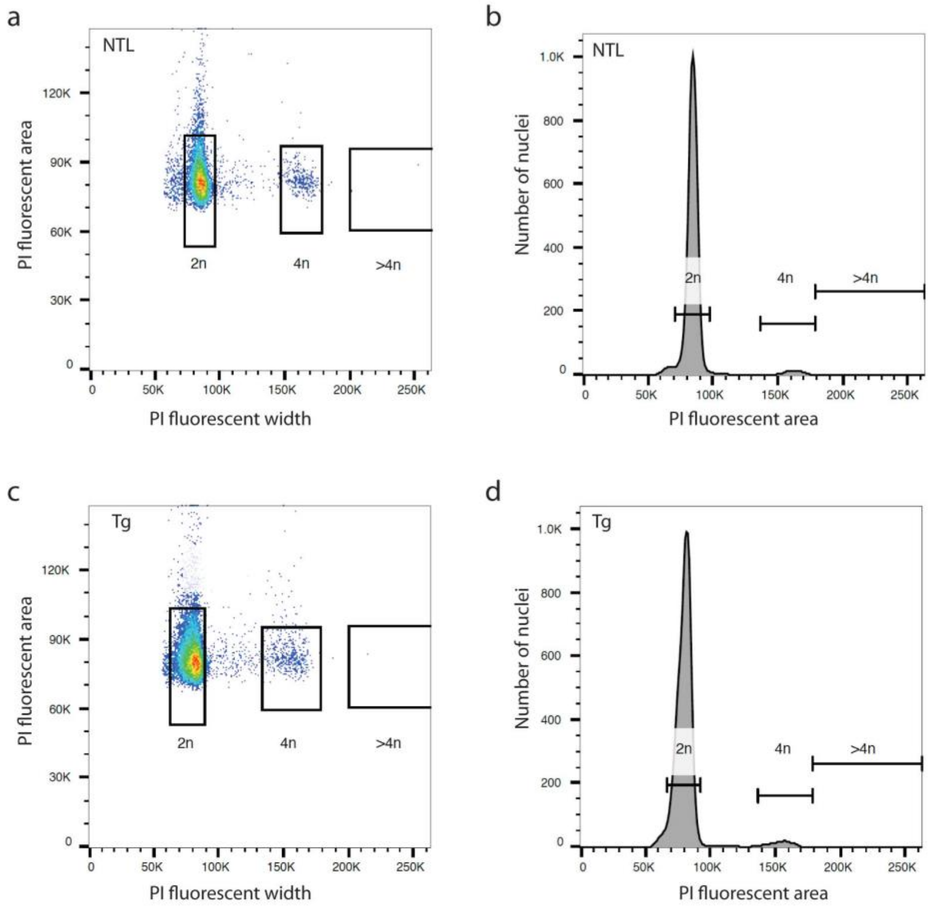

Supplementary Figure S1: Ploidy of CMs isolated from NTL or dn-c-kit-Tg (Tg) hearts is equivalent. (**a, c**) Two-parametric flow cytometry acquisition dot plots showing three distinct subpopulations of nuclei from NTL (**a**) or Tg (**c**) CMs, corresponding to diploid (2n), tetraploid (4n) or >4n nuclei. (**b, d**) Acquisition histograms of nuclei from NTL (**b**) or Tg (**d**) CMs showing three major peaks corresponding to the three different subpopulations in the respective acquisition dot plots (**a, c**). Plots are representative examples from one of three experiments analysing NTL and Tg CMs from *n=4* and *n=6* mice, respectively. PI, propidium iodide.

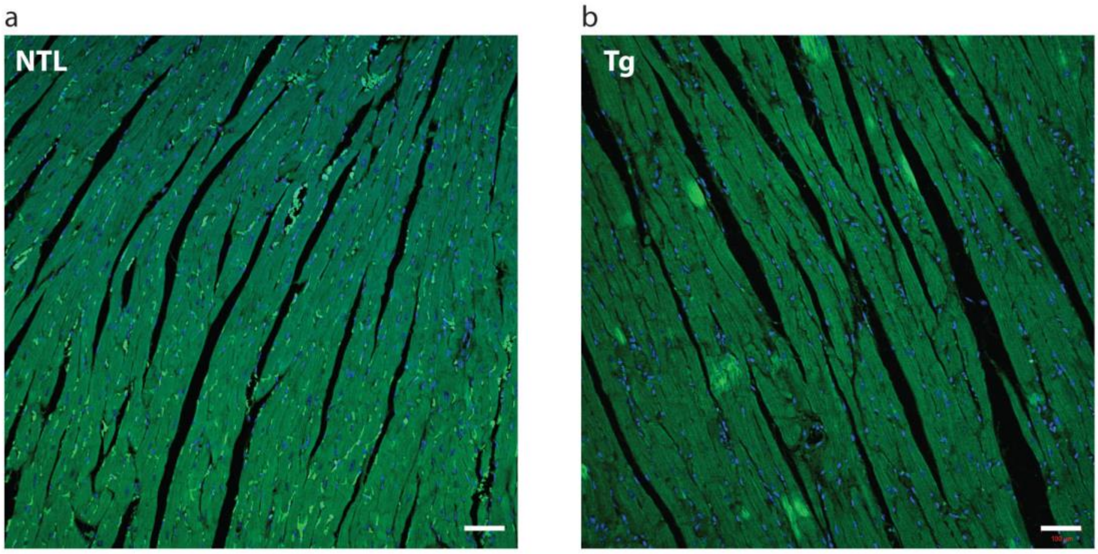

Supplementary Figure S2: No evidence of myofibrillar disarray or CM apoptosis in P112 NTL or dn-c-kit-Tg (Tg) hearts. Representative confocal microscopy images of LV sections from P112 NTL (**a**) or Tg (**b**) hearts stained for DNA strand breaks (red) followed by cTnT (green) and DAPI (blue) revealed no evidence of myofibrillar disarray or CM apoptosis. Images representative of *n=3* mice per genotype. Scale bar: 100 μm.

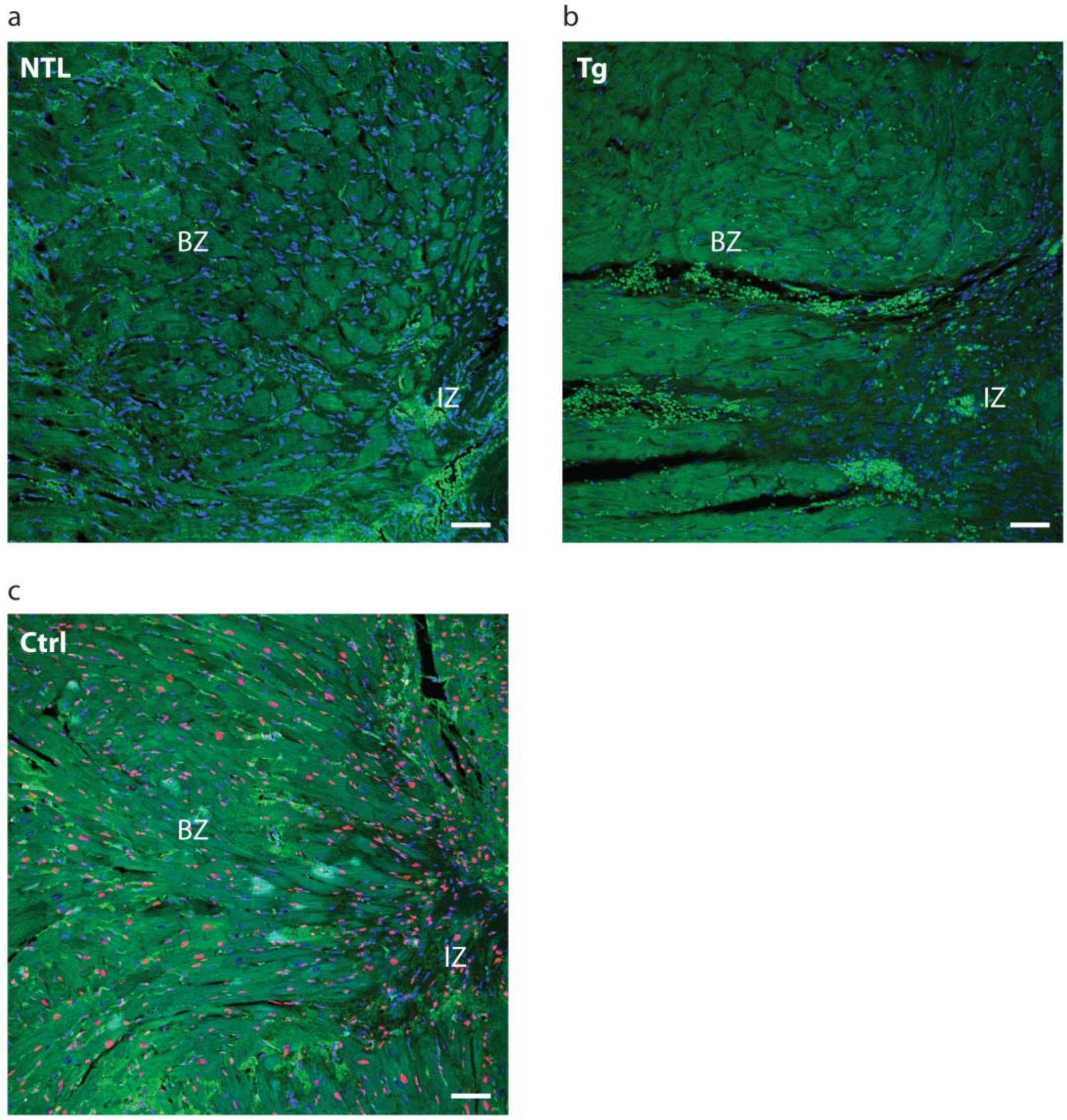

Supplementary Figure S3: No evidence of CM apoptosis in NTL or dn-c-kit-Tg (Tg) hearts 12 weeks post-MI. Representative confocal microscopy images of infarct (IZ) and border zone (BZ) regions of LV from NTL (**a**) or Tg (**b**) hearts 12 weeks post-MI stained for DNA strand breaks (red) followed by cTnT (green) and DAPI (blue) revealed no evidence of CM apoptosis. Images representative of *n=3* mice per genotype. (**c**) DNAse-treated apoptosis positive control of IZ and BZ regions of LV from Tg heart 12 weeks post-MI. Scale bar: 50 μm.

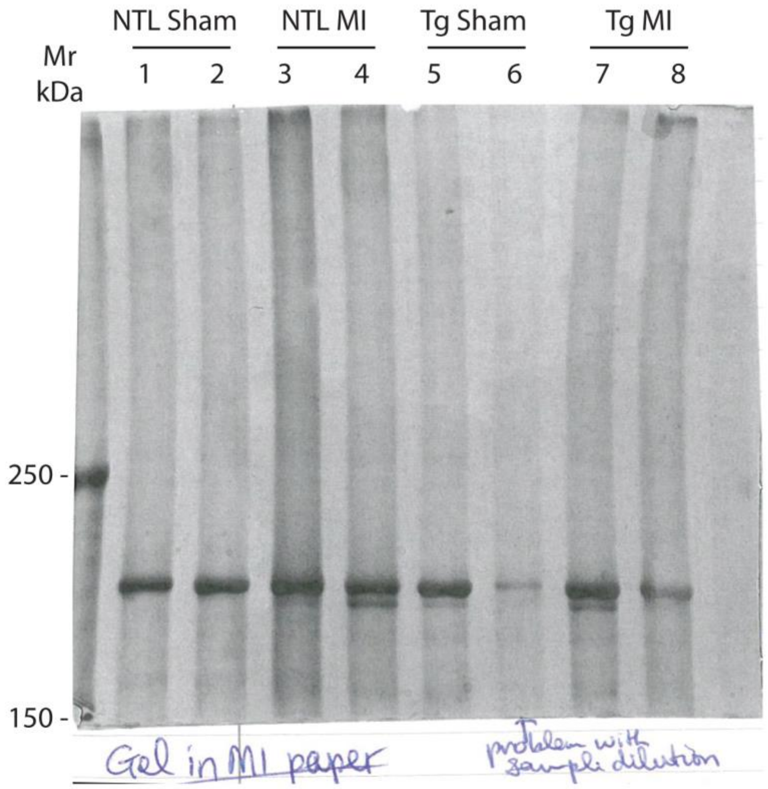

Supplementary Figure S4: Full-length gel from Figure 2f. Silver-stained 6% SDS-polyacrylamide gel of size-fractionated heart homogenates showing the presence of only α-MHC in NTL sham heart, and both α- and β-MHC isoforms in NTL hearts post-MI and in dn-c-kit-Tg (Tg) sham and post-MI hearts (age P196). Lanes 2, 4-5, and 7 were cropped to compile Figure 2f. No adjustments in contrast were made in the preparation of Figure 2f.
